# Crystal structure, Hirshfeld surface analysis and inter­action energy and DFT studies of 2-(2,3-di­hydro-1*H*-perimidin-2-yl)-6-meth­oxy­phenol

**DOI:** 10.1107/S2056989020004284

**Published:** 2020-04-03

**Authors:** Ballo Daouda, Nanou Tiéba Tuo, Tuncer Hökelek, Kangah Niameke Jean-Baptiste, Kodjo Charles Guillaume, Kablan Ahmont Landry Claude, El Mokhtar Essassi

**Affiliations:** a Laboratoire de Chimie Organique Heterocyclique URAC 21, Pôle de Competence Pharmacochimie, Faculté des Sciences, Universite Mohammed V, Rabat, Morocco; b Laboratoire de Chimie Organique et de Substances Naturelles, UFR Sciences des Structures de la Matière et Technologie, Université Félix Houphouët-Boigny, 22 BP 582 Abidjan, Côte d’Ivoire; c Department of Physics, Hacettepe University, 06800 Beytepe, Ankara, Turkey; d Laboratoire de Thermodynamique et Physicochimie du Milieu, Université Nangui Abrogoua, UFR-SFA, 02 BP 801 Abidjan 02, Côte d’Ivoire; e Laboratoire de Cristallographie et Physique Moléculaire, UFR SSMT, Université Félix Houphouët Boigny, 01 BP V34 Abidjan 01, Côte d’Ivoire; f UFR des Sciences Biologiques, Université Péléforo Gon Coulibaly, BP 1328 Korhogo, Côte d’Ivoire

**Keywords:** crystal structure, perimidin, meth­oxy­phenol, Hirshfeld surface

## Abstract

The title compound, C_18_H_16_N_2_O_2_, consists of perimidine and meth­oxy­phenol units, where the tricyclic perimidine unit contains a naphthalene ring system and a non-planar C_4_N_2_ ring adopting an envelope conformation with the NCN group hinged by 47.44 (7)° with respect to the best plane of the other five atoms. In the crystal, O—H_Phnl_⋯N_Prmdn_ and N—H_Prmdn_⋯O_Phnl_ (Phnl = phenol and Prmdn = perimidine) hydrogen bonds link the mol­ecules into infinite chains along the *b*-axis direction. Weak C—H⋯π inter­actions may further stabilize the crystal structure. The Hirshfeld surface analysis of the crystal structure indicates that the most important contributions for the crystal packing are from H⋯H (49.0%), H⋯C/C⋯H (35.8%) and H⋯O/O⋯H (12.0%) inter­actions. Hydrogen bonding and van der Waals inter­actions are the dominant inter­actions in the crystal packing. Computational chemistry indicates that in the crystal, the O—H_Phnl_⋯N_Prmdn_ and N—H_Prmdn_⋯O_Phnl_ hydrogen-bond energies are 58.4 and 38.0 kJ mol^−1^, respectively. Density functional theory (DFT) optimized structures at the B3LYP/ 6–311 G(d,p) level are compared with the experimentally determined mol­ecular structure in the solid state. The HOMO–LUMO behaviour was elucidated to determine the energy gap.

## Abstract 2

The title compound consists of perimidin and meth­oxy­phenol units. In the crystal, O—H_Phnl_⋯N_Prmdn_ and N—H_Prmdn_⋯O_Phnl_ (Phnl = phenol and Prmdn = perimidine) hydrogen bonds link the mol­ecules into infinite chains along the *b*-axis direction. C—H⋯π inter­actions may further stabilize the crystal structure.

## Chemical context   

Six-membered heterocyclic compounds carrying two nitro­gen atoms have been widely studied (Aly & El-Shaieb, 2004 ▸; Koca *et al.*, 2012 ▸; Zhao *et al.*, 2012 ▸; Baranov & Fadeev, 2016 ▸; Lahmidi *et al.*, 2018 ▸). Perimidine derivatives (peri­naphtho-fused perimidine ring systems) in particular have aroused a lot of inter­est because of their applications in photophysics (Del Valle *et al.*, 1997 ▸) and their use as colouring matters for polyester fibers (Claramunt *et al.*, 1995 ▸) and as fluorescent materials (Varsha *et al.*, 2010 ▸). These mol­ecules have a wide range of biological applications (Dzieduszycka *et al.*, 2002 ▸), indicating that the perimidine group is a potentially useful model in medicinal chemistry research and therapeutic applications. In coordination chemistry, perimidine derivatives have been studied for their inter­esting catalytic activities (Cucciolito *et al.*, 2013*a* ▸; Akıncı *et al.*, 2014 ▸) as well as in the field of corrosion inhibition (He *et al.*, 2018 ▸). As a continuation of our research on the development of new perimidine derivatives with potential pharmacological applications, we studied the condensation reaction of *ortho*-vanillin and 1,8-di­aminona­phthalene in ether under agitation at room temperature, which gave the title compound, 2-(2,3-di­hydro-1*H*-perimidin-2-yl)-6-meth­oxy­phenol, in good yield. We report herein the synthesis, the mol­ecular and crystal structures along with Hirshfeld surface analysis and computational calculations of the title compound, (I).
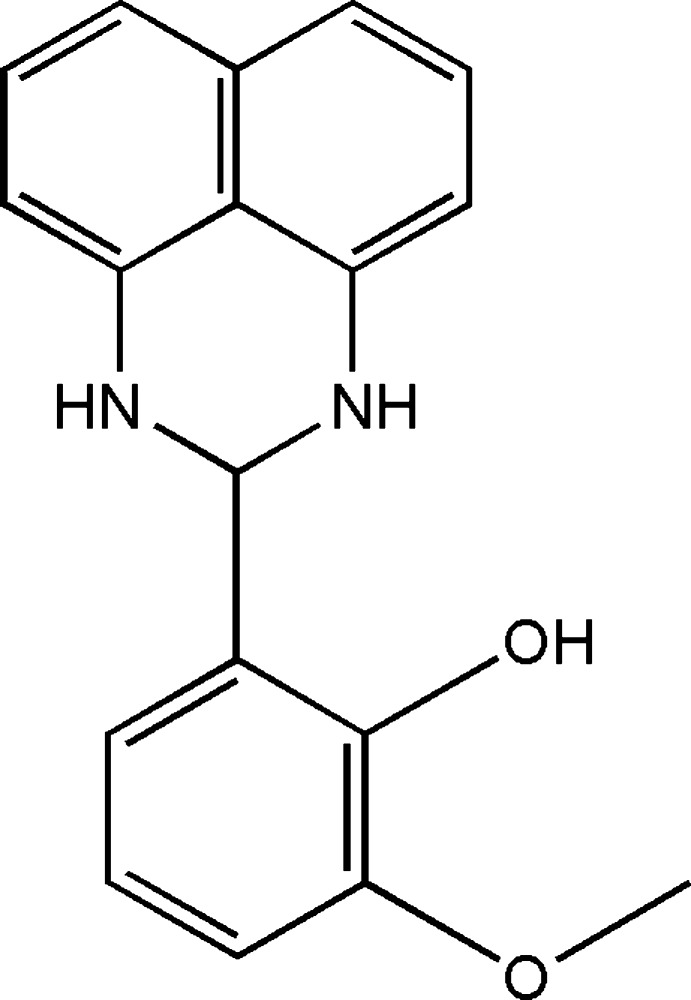


## Structural commentary   

**Figure 1:**
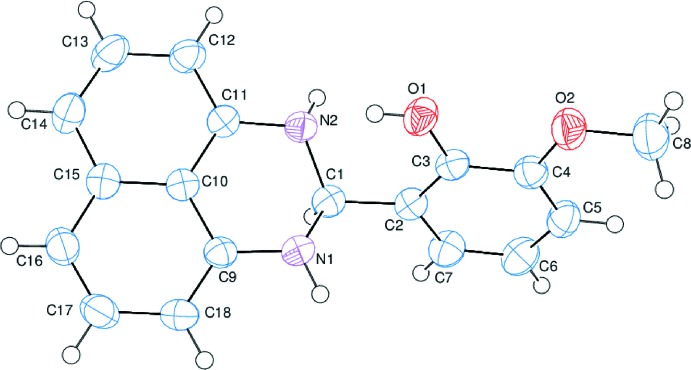
The asymmetric unit of the title compound with the atom-numbering scheme. Displacement ellipsoids are drawn at the 50% probability level.

The title compound, (I), consists of perimidine and meth­oxy­phenol units, where the tricyclic perimidine unit contains a naphthalene ring system and a non-planar C_4_N_2_ ring (Fig. 1 ▸). A puckering analysis of the non-planar six-membered C_4_N_2_, *B* (N1/N2/C1/C9–C11), ring gave the parameters *q*_2_ = 0.3879 (12) Å, *q*_3_ = −0.2565 (12) Å, *Q*_T_ = 0.4650 (13) Å, θ_2_ = 123.47 (15)° and φ_2_ = 235.98 (18)°]. The ring adopts an envelope conformation, where atom C1 is at the flap position and at a distance of 0.6454 (12) Å from the best plane through the other five atoms. The C_4_N_2_ ring is hinged about the N⋯N vector with the N1—C1—N2 plane being inclined by 47.44 (7)° to the best plane of the other five atoms (N1/N2/C9–C11). In the meth­oxy­phenol moiety, the C8—O2—C4—C5 and C8—O2—C4—C3 torsion angles are −2.9 (2)° and 176.72 (12)°, respectively. Rings *A* (C2–C7), *C* (C10–C15) and *D* (C9/C10/C15–C18) are oriented at dihedral angles of *A*/*C* = 65.39 (4)°, *A*/*D* = 69.63 (4)° and *C*/*D* = 4.31 (3)°.

## Supra­molecular features   

**Figure 2:**
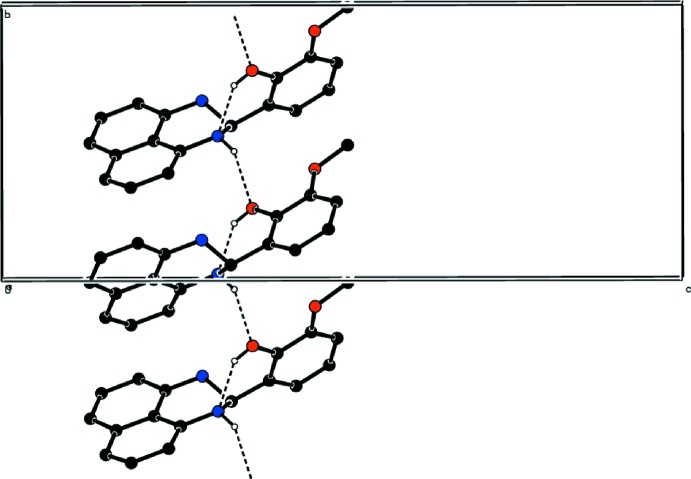
A partial packing diagram viewed along the *a*-axis direction with O—H_Phnl_⋯N_Prmdn_ and N—H_Prmdn_⋯O_Phnl_ (Phnl = phenol and Prmdn = perimidine) hydrogen bonds shown as dashed lines. H-atoms not included in hydrogen bonding have been omitted for clarity.

In the crystal, O—H_Phnl_⋯N_Prmdn_ and N—H_Prmdn_⋯O_Phnl_ (Phnl = phenol and Prmdn = perimidine) hydrogen bonds (Table 1 ▸) link the mol­ecules into infinite chains along the *b*-axis direction (Fig. 2 ▸). The C—H⋯π inter­actions (Table 1 ▸) may further stabilize the crystal structure.

## Hirshfeld surface analysis   

**Figure 3:**
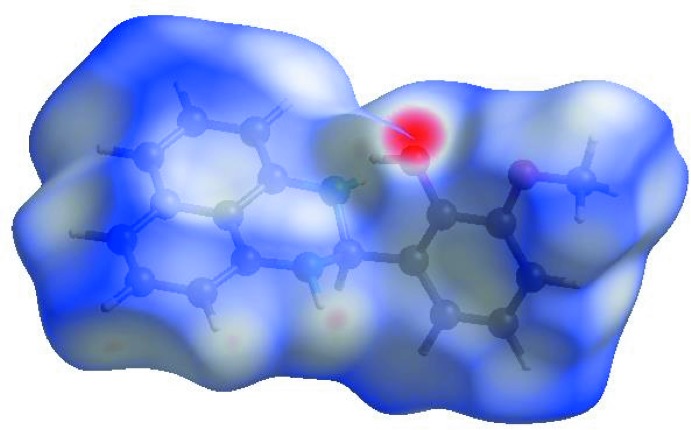
View of the three-dimensional Hirshfeld surface of the title compound plotted over *d*_norm_ in the range −0.4133 to 1.3883 a.u.

**Figure 4:**
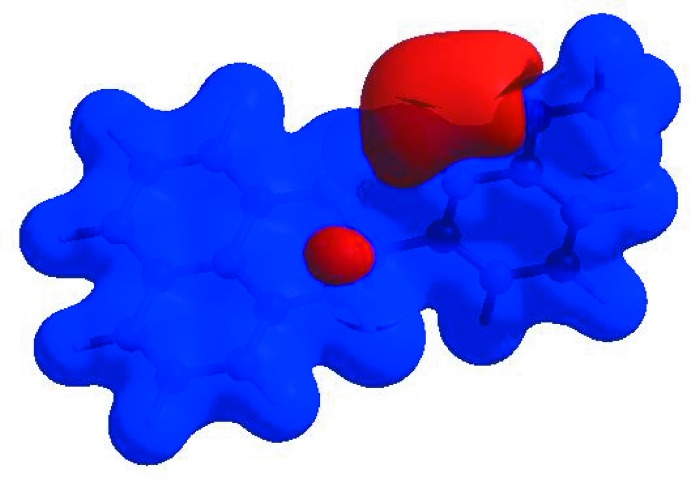
View of the three-dimensional Hirshfeld surface of the title compound plotted over electrostatic potential energy in the range −0.0500 to 0.0500 a.u. using the STO-3 G basis set at the Hartree–Fock level of theory. Hydrogen-bond donors and acceptors are shown as blue and red regions around the atoms corresponding to positive and negative potentials, respectively.

**Figure 5:**
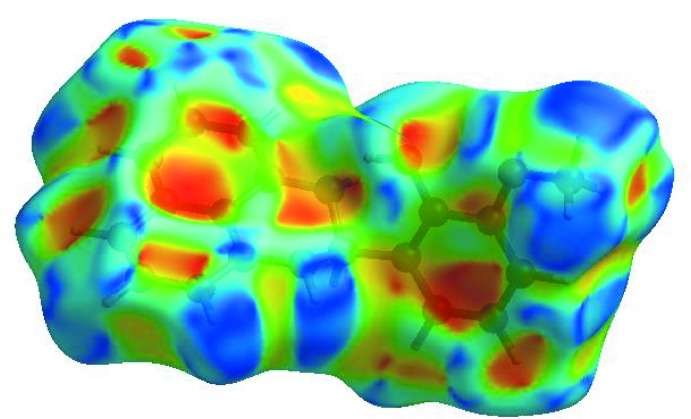
Hirshfeld surface of the title compound plotted over shape-index.

In order to visualize the inter­molecular inter­actions in the crystal of the title compound, a Hirshfeld surface (HS) analysis (Hirshfeld, 1977 ▸; Spackman & Jayatilaka, 2009 ▸) was carried out by using *Crystal Explorer 17.5* (Turner *et al.*, 2017 ▸). In the HS plotted over *d*_norm_ (Fig. 3 ▸), the white surface indicates contacts with distances equal to the sum of van der Waals radii, and the red and blue colours indicate distances shorter (in close contact) or longer (distinct contact) than the van der Waals radii, respectively (Venkatesan *et al.*, 2016 ▸). The bright-red spot appearing near O1 indicates its role as the respective donor and/or acceptor; it also appears as blue and red regions corresponding to positive and negative potentials on the HS mapped over electrostatic potential (Spackman *et al.*, 2008 ▸; Jayatilaka *et al.*, 2005 ▸) as shown in Fig. 4 ▸. The blue regions indicate the positive electrostatic potential (hydrogen-bond donors), while the red regions indicate the negative electrostatic potential (hydrogen-bond acceptors). The shape-index of the HS is a tool to visualize the π–π stacking by the presence of adjacent red and blue triangles; if there are no adjacent red and/or blue triangles, then there are no π–π inter­actions. Fig. 5 ▸ clearly suggests that there are no π–π inter­actions in (I).

**Figure 6:**
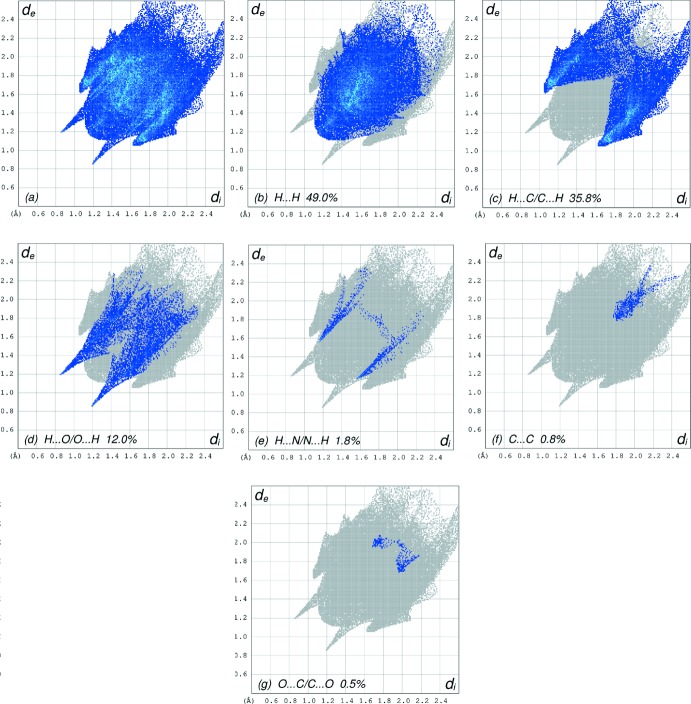
The full two-dimensional fingerprint plots for the title compound, showing (*a*) all inter­actions, and delineated into (*b*) H⋯H, (*c*) H⋯C/C⋯H, (*d*) H⋯O/O⋯H, (*e*) H⋯N/N⋯H and (*f*) O⋯C/C⋯O inter­actions. The *d*_i_ and *d*_e_ values are the closest inter­nal and external distances (in Å) from given points on the Hirshfeld surface contacts.

The overall two-dimensional fingerprint plot, Fig. 6 ▸ *a*, and those delineated into H⋯H, H⋯C/C⋯H, H⋯O/O⋯H, H⋯N/N⋯H, C⋯C and O⋯C/C⋯O contacts (McKinnon *et al.*, 2007 ▸) are illustrated in Fig. 6 ▸ *b*-*g*, respectively, together with their relative contributions to the Hirshfeld surface. The most important inter­action is H⋯H contributing 49.0% to the overall crystal packing, which is reflected in Fig. 6 ▸ *b* as widely scattered points of high density due to the large hydrogen-atom content of the mol­ecule with the tip at *d*_e_ = *d*_i_ = 1.20 Å. In the presence of C—H⋯π inter­actions, the pair of characteristic wings in the fingerprint plot, Fig. 6 ▸ *c*, delineated into H⋯C/C⋯H contacts (Table 2 ▸; 35.8% contribution to the HS) have the tips at *d*_e_ + *d*_i_ = 2.68 Å. The pair of spikes in the fingerprint plot delineated into H⋯O/O⋯H contacts (12.0% contribution, Fig. 6 ▸ *d*) have a symmetrical distribution of points with the tips at *d*_e_ + *d*_i_ = 3.03 Å. The H⋯N/N⋯H contacts (Fig. 6 ▸ *e*, 1.8% contribution) have a distribution of points with the tips at *d*_e_ + *d*_i_ = 2.72 Å. The C⋯C contacts (0.8%, Fig. 6 ▸ *f*) have the tip at *d*_e_ = *d*_i_ = 3.37 Å. Finally, the O⋯C/C⋯O inter­actions make only a 0.5% contribution to the overall crystal packing.

**Figure 7:**
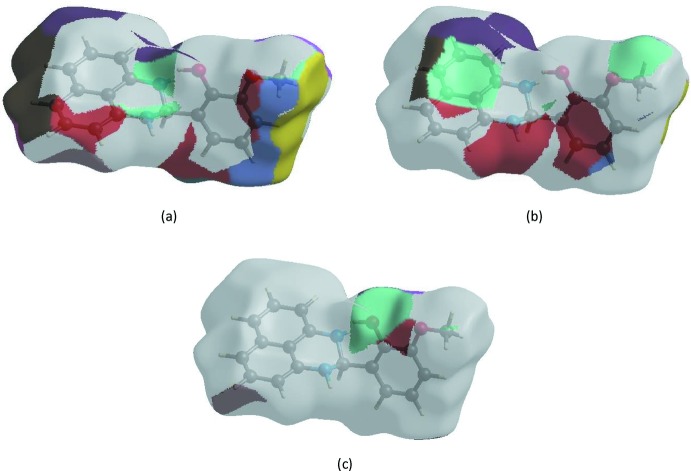
The Hirshfeld surface representations with the function *d*_norm_ plotted onto the surface for (*a*) H⋯H, (*b*) H⋯C/C⋯H and (*c*) H⋯O/O⋯H inter­actions.

The Hirshfeld surface representations with the function *d*_norm_ plotted onto the surface are shown for the H⋯H, H⋯C/C⋯H and H⋯O/O⋯H inter­actions in Fig. 7 ▸ *a*–*c*, respectively.

The Hirshfeld surface analysis confirms the importance of H-atom contacts in establishing the packing. The large number of H⋯H, H⋯C/C⋯H and H⋯O/O⋯H inter­actions suggest that van der Waals inter­actions and hydrogen bonding play the major roles in the crystal packing (Hathwar *et al.*, 2015 ▸).

## Inter­action energy calculations   

The inter­molecular inter­action energies were calculated using the CE–B3LYP/6–31G(d,p) energy model available in *Crystal Explorer 17.5* (Turner *et al.*, 2017 ▸), where a cluster of mol­ecules is generated by applying crystallographic symmetry operations with respect to a selected central mol­ecule within a default radius of 3.8 Å (Turner *et al.*, 2014 ▸). The total inter­molecular energy (*E*_tot_) is the sum of electrostatic (*E*_ele_), polarization (*E*_pol_), dispersion (*E*_dis_) and exchange-repulsion (*E*_rep_) energies (Turner *et al.*, 2015 ▸) with scale factors of 1.057, 0.740, 0.871 and 0.618, respectively (Mackenzie *et al.*, 2017 ▸). Hydrogen-bonding inter­action energies (in kJ mol^−1^) were calculated as −37.5 (*E*_ele_), −7.8 (*E*_pol_), −52.0 (*E*_dis_), 52.4 (*E*_rep_) and −58.4 (*E*_tot_) [or O1—H1*O*⋯N2 and −11.3 (*E*_ele_), −3.4 (*E*_pol_), −48.4 (*E*_dis_), 30.0 (*E*_rep_) and −38.0 (*E*_tot_) for N2—H2*N*⋯O1.

## DFT calculations   

**Figure 8:**
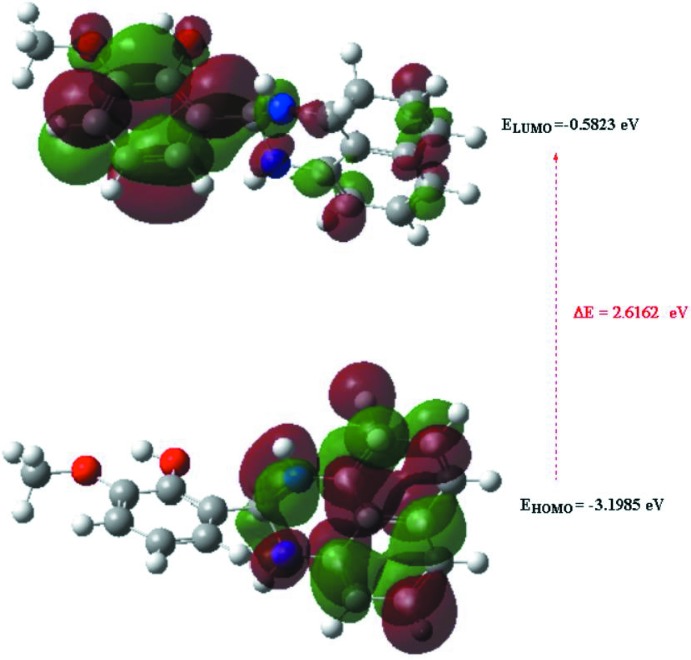
The energy band gap of the title compound, (I).

The optimized structure of the title compound, (I), in the gas phase was generated theoretically *via* density functional theory (DFT) using standard B3LYP functional and 6–311 G(d,p) basis-set calculations (Becke, 1993 ▸) as implemented in *GAUSSIAN 09* (Frisch *et al.*, 2009 ▸). The theoretical and experimental results were in good agreement (Table 3 ▸). The highest-occupied mol­ecular orbital (HOMO), acting as an electron donor, and the lowest-unoccupied mol­ecular orbital (LUMO), acting as an electron acceptor, are very important parameters for quantum chemistry. When the energy gap is small, the mol­ecule is highly polarizable and has high chemical reactivity. The DFT calculations provide some important information on the reactivity and site selectivity of the mol­ecular framework. *E*_HOMO_ and *E*_LUMO_ clarify the inevitable charge-exchange collaboration inside the studied material, electronegativity (χ), hardness (η), potential (μ), electrophilicity (ω) and softness (*σ*) are recorded in Table 4 ▸. The significance of η and *σ* is for the evaluation of both the reactivity and stability. The electron transition from the HOMO to the LUMO energy level is shown in Fig. 8 ▸. The HOMO and LUMO are localized in the plane extending from the whole 2-(2,3-di­hydro-1*H*-perimidin-2-yl)-6-meth­oxy­phenol ring. The energy band gap [Δ*E* = *E*_LUMO_ − *E*_HOMO_] of the mol­ecule is about 2.6162 eV, and the frontier mol­ecular orbital energies, *E*_HOMO_ and *E*_LUMO_ are −3.1985 and −0.5823 eV, respectively.

## Database survey   

Similar compounds of the perimidine derivative have also been reported (Ghorbani, 2012 ▸; Fun *et al.*, 2011 ▸; Maloney *et al.*, 2013 ▸; Cucciolito *et al.*, 2013*b* ▸; Manimekalai *et al.*, 2014 ▸), in which the groups at position 2 are almost coplanar with the perimidic nucleus (Ghorbani, 2012 ▸; Fun *et al.*, 2011 ▸; Cucciolito *et al.*, 2013*b* ▸). The closest examples to the title compound, (I), are (II) (Cucciolito *et al.*, 2013*b* ▸) and (III) (Fun *et al.*, 2011 ▸), while (IV) (Ghorbani, 2012 ▸), (V) (Maloney *et al.*, 2013 ▸) and (VI) (Manimekalai *et al.*, 2014 ▸) are more distant relatives.
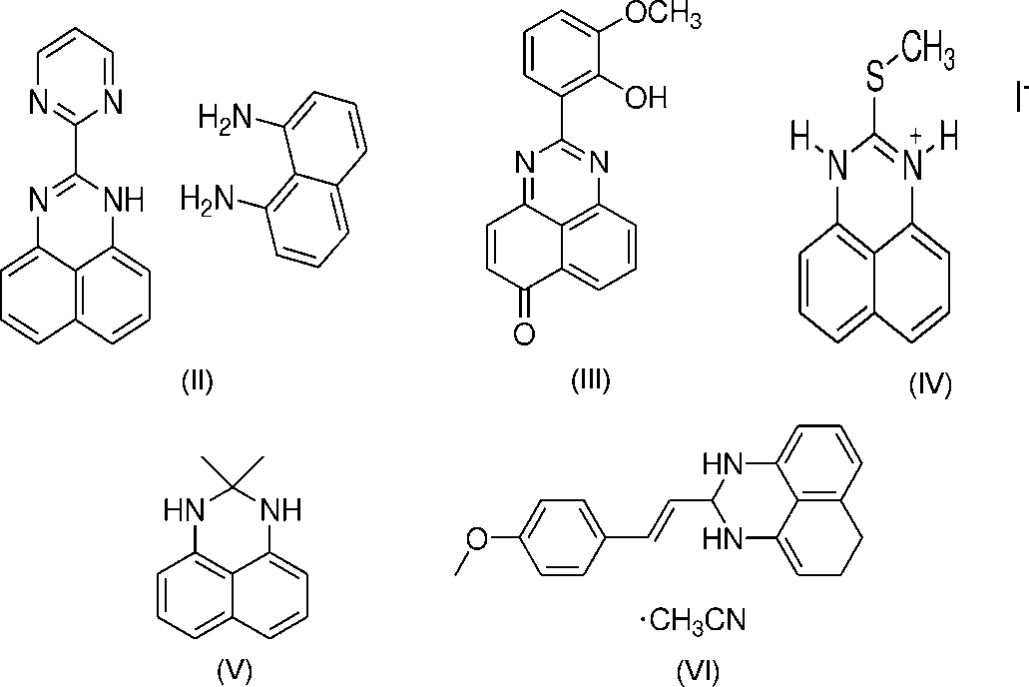


## Synthesis and crystallization   

The title compound, (I), was synthesized from the condensation of *ortho*-vanillin (3 mmol) and 1,8- di­aminona­phthalene (4 mmol) in ether (30 ml) under agitation at room temperature. Brown single crystals were obtained by the slow evaporation of the acetone solvent after 15 days (yield: 75%).

## Refinement   

Crystal data, data collection and structure refinement details are summarized in Table 5 ▸. The C-bound H atoms were positioned geometrically, with C—H = 0.93 Å (for aromatic H atoms and H14*C*, H15*A* and H15*B* of the allyl moiety), 0.98 Å (for methine H atom) and 0.97 Å (for methyl­ene H atoms), and constrained to ride on their parent atoms, with *U*_iso_(H) = 1.2*U*_eq_(C). The hydroxyl H atom was placed in a calculated position with O—H = 0.82 Å and *U*_iso_(H) = 1.5*U*_eq_(O) while H atoms bonded to N atoms were refined independently with *U*_iso_(H) = 1.2*U*_eq_(N)

## Supplementary Material

Crystal structure: contains datablock(s) I, global. DOI: 10.1107/S2056989020004284/lh5952sup1.cif

Structure factors: contains datablock(s) I. DOI: 10.1107/S2056989020004284/lh5952Isup2.hkl

Supporting information file. DOI: 10.1107/S2056989020004284/lh5952Isup3.cdx

Supporting information file. DOI: 10.1107/S2056989020004284/lh5952Isup4.cml

CCDC reference: 1976883

Additional supporting information:  crystallographic information; 3D view; checkCIF report

## Figures and Tables

**Table 1:** Hydrogen-bond geometry (Å, °) *Cg*1 and *Cg*4 are the centroids of rings *A* (C2–C7) and *D* (C9/C10/C15–C18), respectively.

*D*—H⋯*A*	*D*—H	H⋯*A*	*D*⋯*A*	*D*—H⋯*A*
O1—H1*O*⋯N2	0.82	1.96	2.6667 (14)	144
N2—H2*N*⋯O1^i^	0.864 (15)	2.196 (15)	2.9870 (14)	152.2 (13)
C8—H8*A*⋯*Cg*1^iv^	0.96	2.82	3.6580 (17)	146
C13—H13⋯*Cg*4^i^	0.93	2.87	3.7336 (16)	155
C16—H16⋯*Cg*1^v^	0.93	2.87	3.4880 (15)	125

Symmetry codes: (i) 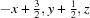; (iv) 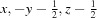; (v) 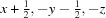.

**Table 2:** Selected interatomic distances (Å)

O1⋯O2	2.5772 (14)	C5⋯H8*A* ^i^	2.94
O1⋯N2	2.6668 (14)	C5⋯H8*B*	2.72
C12⋯O1^i^	3.1736 (17)	C5⋯H8*C*	2.80
C17⋯O1^ii^	3.3145 (17)	C8⋯H5	2.55
C11⋯O1^i^	3.3650 (15)	H13⋯C9^i^	2.93
N2⋯O1^i^	2.9867 (14)	H13⋯C10^i^	2.97
H2*N*⋯O1^i^	2.196 (15)	C10⋯H1	2.95
H18⋯O1^ii^	2.88	C12⋯H1*O* ^i^	2.88
H12⋯O1^i^	2.66	H1⋯H7	2.39
H17⋯O1^ii^	2.63	H1*N*⋯H18	2.44
O2⋯H6^iii^	2.87	H1*O*⋯H2*N*	2.31
N1⋯H1*O*	2.86	H17⋯H1*O* ^ii^	2.57
H12⋯N1^i^	2.86	H18⋯H1*O* ^ii^	2.47
N2⋯H1*O*	1.96	H2*N*⋯H12	2.43
C18⋯C12^ii^	3.567 (2)	H5⋯H8*B*	2.28
C1⋯H1*O*	2.46	H5⋯H8*C*	2.40
C4⋯H8*A* ^i^	2.92	H14⋯H16	2.53

Symmetry codes: (i) 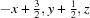; (ii) 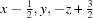; (iii) 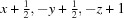.

**Table 3:** Comparison of selected (X-ray and DFT) geometric data (Å, °)

Bonds/angles	X-ray	B3LYP/6–311G(d,p)
O1—C3	1.3587 (14)	1.38948
O1—H1*O* ^*a*^	0.82	0.97611
N1—C9	1.3865 (16)	1.39921
N1—C1	1.4529 (17)	1.47118
N1—H1*N*	0.870 (15)	0.90721
C1—N2	1.4745 (16)	1.47531
C1—C2	1.5074 (17)	1.51309
O2—C4	1.3677 (15)	1.40231
O2—C8	1.4088 (17)	1.45201
N2—C11	1.4124 (16)	1.39016
N2—H2*N*	0.864 (15)	0.90717
		
C3—O1—H1*O* ^*a*^	109.5	109.04
C9—N1—C1	116.86 (10)	117.19
C9—N1—H1*N*	115.4 (10)	116.29
C1—N1—H1*N*	113.3 (10)	114.01
N1—C1—N2	106.56 (10)	106.87
N1—C1—C2	109.17 (10)	110.78
N2—C1—C2	111.38 (10)	110.82
N1—C1—H1^*a*^	109.9	110.12
N2—C1—H1^*a*^	109.9	109.09

Note: (*a*) These four entries were not refined, as they each include a constrained H atom.

**Table 4:** Calculated energies

Mol­ecular Energy (a.u.) (eV)	Compound (I)
Total Energy *TE* (eV)	−26013
*E*_HOMO_ (eV)	−3.1985
*E*_LUMO_ (eV)	−0.5823
Gap, *ΔE* (eV)	2.6162
Dipole moment, *μ* (Debye)	7.0880
Ionization potential, *I* (eV)	3.1985
Electron affinity, *A*	0.5823
Electronegativity, *χ*	1.8904
Hardness, *η*	1.3081
Electrophilicity index, *ω*	1.3660
Softness, *σ*	0.7645
Fraction of electrons transferred, *ΔN*	1.9530

**Table 5:** Experimental details

Crystal data	
Chemical formula	C_18_H_16_N_2_O_2_
*M*_r_	292.33
Crystal system, space group	Orthorhombic, *P* *b* *c* *a*
Temperature (K)	293
*a*, *b*, *c* (Å)	12.7245 (7), 9.5887 (6), 23.7276 (14)
*V* (Å^3^)	2895.0 (3)
*Z*	8
Radiation type	Mo *K*α
μ (mm^−1^)	0.09
Crystal size (mm)	0.52 × 0.10 × 0.04

Data collection	
Diffractometer	Rigaku XtaLAB PRO
Absorption correction	Multi-scan (*CrysAlis PRO*; Rigaku OD, 2018 ▸)
*T*_min_, *T*_max_	0.390, 1.000
No. of measured, independent and observed [*I* > 2σ(*I*)] reflections	16885, 3499, 2640
*R*_int_	0.036
(sin θ/λ)_max_ (Å^−1^)	0.682

Refinement	
*R*[*F* ^2^ > 2σ(*F* ^2^)], *wR*(*F* ^2^), *S*	0.045, 0.118, 1.04
No. of reflections	3496
No. of parameters	207
H-atom treatment	H atoms treated by a mixture of independent and constrained refinement
Δρ_max_, Δρ_min_ (e Å^−3^)	0.20, −0.21

Computer programs: *CrysAlis PRO* (Rigaku OD, 2018 ▸), *SHELXT* (Sheldrick, 2015*a* ▸) and *SHELXL2018/3* (Sheldrick, 2015*b* ▸).

## supplementary crystallographic information

### Crystal data

**Table:**

C_18_H_16_N_2_O_2_	*D*_x_ = 1.341 Mg m^−^^3^
*M**_r_* = 292.33	Mo *K*α radiation, λ = 0.71073 Å
Orthorhombic, *P**b**c**a*	Cell parameters from 6789 reflections
*a* = 12.7245 (7) Å	θ = 3.2–28.1°
*b* = 9.5887 (6) Å	µ = 0.09 mm^−^^1^
*c* = 23.7276 (14) Å	*T* = 293 K
*V* = 2895.0 (3) Å^3^	Elongated platelet, brown
*Z* = 8	0.52 × 0.10 × 0.04 mm
*F*(000) = 1232	

### Data collection

**Table:**

Rigaku XtaLAB PRO diffractometer	3499 independent reflections
Radiation source: micro-focus sealed X-ray tube, Rigaku micromax 003	2640 reflections with *I* > 2σ(*I*)
Rigaku Integrated Confocal MaxFlux double bounce multi-layer mirror optics monochromator	*R*_int_ = 0.036
Detector resolution: 5.811 pixels mm^-1^	θ_max_ = 29.0°, θ_min_ = 2.4°
ω scans	*h* = −16→14
Absorption correction: multi-scan (CrysAlisPro; Rigaku OD, 2018)	*k* = −12→13
*T*_min_ = 0.390, *T*_max_ = 1.000	*l* = −30→30
16885 measured reflections	

### Refinement

**Table:**

Refinement on *F*^2^	Primary atom site location: other
Least-squares matrix: full	Secondary atom site location: difference Fourier map
*R*[*F*^2^ > 2σ(*F*^2^)] = 0.045	Hydrogen site location: mixed
*wR*(*F*^2^) = 0.118	H atoms treated by a mixture of independent and constrained refinement
*S* = 1.04	*w* = 1/[σ^2^(*F*_o_^2^) + (0.0609*P*)^2^ + 0.4131*P*] where *P* = (*F*_o_^2^ + 2*F*_c_^2^)/3
3496 reflections	(Δ/σ)_max_ = 0.001
207 parameters	Δρ_max_ = 0.20 e Å^−^^3^
0 restraints	Δρ_min_ = −0.21 e Å^−^^3^

### Special details

**Table:**

Geometry. All esds (except the esd in the dihedral angle between two l.s. planes) are estimated using the full covariance matrix. The cell esds are taken into account individually in the estimation of esds in distances, angles and torsion angles; correlations between esds in cell parameters are only used when they are defined by crystal symmetry. An approximate (isotropic) treatment of cell esds is used for estimating esds involving l.s. planes.

### Fractional atomic coordinates and isotropic or equivalent isotropic displacement parameters (Å^2^)

**Table:**

	*x*	*y*	*z*	*U*_iso_*/*U*_eq_	
O1	0.73352 (7)	0.23966 (10)	0.63178 (4)	0.0437 (2)	
H1O	0.731483	0.294081	0.658419	0.066*	
N1	0.52935 (9)	0.35194 (11)	0.70590 (5)	0.0395 (3)	
H1N	0.4730 (12)	0.3089 (15)	0.6946 (6)	0.047*	
C1	0.57436 (10)	0.44289 (13)	0.66329 (5)	0.0373 (3)	
H1	0.532216	0.528041	0.659972	0.045*	
O2	0.72915 (8)	0.09678 (11)	0.53981 (4)	0.0519 (3)	
N2	0.68133 (8)	0.47793 (11)	0.68254 (4)	0.0379 (3)	
H2N	0.7113 (11)	0.5331 (16)	0.6587 (6)	0.045*	
C2	0.57650 (10)	0.36729 (13)	0.60760 (5)	0.0363 (3)	
C3	0.65312 (9)	0.26779 (12)	0.59586 (5)	0.0346 (3)	
C4	0.64940 (10)	0.19120 (13)	0.54596 (5)	0.0383 (3)	
C5	0.56894 (11)	0.21405 (14)	0.50790 (6)	0.0449 (3)	
H5	0.566134	0.163263	0.474546	0.054*	
C6	0.49246 (11)	0.31282 (15)	0.51959 (6)	0.0500 (4)	
H6	0.438253	0.327981	0.494027	0.060*	
C9	0.52580 (9)	0.40296 (12)	0.76054 (5)	0.0346 (3)	
C7	0.49623 (11)	0.38823 (14)	0.56858 (6)	0.0458 (3)	
H7	0.444503	0.454306	0.575894	0.055*	
C8	0.72720 (13)	0.01056 (17)	0.49174 (7)	0.0603 (4)	
H8A	0.788077	−0.048578	0.491698	0.090*	
H8B	0.727304	0.067394	0.458433	0.090*	
H8C	0.664899	−0.045880	0.492341	0.090*	
C10	0.60767 (9)	0.49588 (12)	0.77664 (5)	0.0335 (3)	
C11	0.68804 (9)	0.53139 (12)	0.73789 (5)	0.0347 (3)	
C12	0.77148 (11)	0.61181 (14)	0.75512 (6)	0.0441 (3)	
H12	0.825905	0.631841	0.730241	0.053*	
C13	0.77415 (12)	0.66349 (15)	0.81036 (6)	0.0506 (4)	
H13	0.830410	0.718819	0.821577	0.061*	
C14	0.69668 (12)	0.63475 (14)	0.84781 (6)	0.0479 (3)	
H14	0.699244	0.673103	0.883784	0.057*	
C15	0.61190 (10)	0.54681 (13)	0.83261 (5)	0.0384 (3)	
C16	0.53296 (10)	0.50546 (15)	0.87065 (6)	0.0451 (3)	
H16	0.532878	0.540825	0.907150	0.054*	
C17	0.45685 (11)	0.41435 (16)	0.85456 (6)	0.0498 (4)	
H17	0.406633	0.386074	0.880644	0.060*	
C18	0.45257 (10)	0.36232 (15)	0.79961 (6)	0.0445 (3)	
H18	0.399877	0.299926	0.789503	0.053*	

### Atomic displacement parameters (Å^2^)

**Table:**

	*U*^11^	*U*^22^	*U*^33^	*U*^12^	*U*^13^	*U*^23^
O1	0.0417 (5)	0.0466 (5)	0.0427 (5)	0.0082 (4)	−0.0125 (4)	−0.0068 (4)
N1	0.0352 (6)	0.0420 (6)	0.0412 (6)	−0.0088 (5)	0.0001 (4)	−0.0046 (5)
C1	0.0358 (7)	0.0346 (6)	0.0416 (7)	0.0020 (5)	−0.0025 (5)	0.0001 (5)
O2	0.0499 (6)	0.0560 (6)	0.0499 (6)	0.0108 (5)	−0.0044 (4)	−0.0146 (5)
N2	0.0378 (6)	0.0389 (6)	0.0370 (6)	−0.0084 (5)	0.0024 (4)	0.0003 (4)
C2	0.0382 (6)	0.0346 (6)	0.0361 (6)	−0.0014 (5)	−0.0029 (5)	0.0039 (5)
C3	0.0336 (6)	0.0356 (6)	0.0347 (6)	−0.0029 (5)	−0.0035 (5)	0.0048 (5)
C4	0.0380 (7)	0.0381 (6)	0.0387 (7)	−0.0015 (5)	0.0007 (5)	0.0012 (5)
C5	0.0510 (8)	0.0482 (8)	0.0354 (7)	−0.0046 (6)	−0.0051 (6)	−0.0013 (6)
C6	0.0500 (8)	0.0550 (8)	0.0451 (8)	0.0023 (7)	−0.0163 (6)	0.0049 (7)
C9	0.0309 (6)	0.0339 (6)	0.0389 (7)	0.0033 (5)	−0.0009 (5)	0.0000 (5)
C7	0.0439 (7)	0.0458 (7)	0.0476 (8)	0.0072 (6)	−0.0095 (6)	0.0021 (6)
C8	0.0658 (10)	0.0599 (10)	0.0551 (9)	0.0081 (8)	0.0010 (7)	−0.0165 (8)
C10	0.0336 (6)	0.0298 (6)	0.0372 (6)	0.0032 (5)	−0.0017 (5)	0.0027 (5)
C11	0.0371 (6)	0.0300 (6)	0.0371 (6)	−0.0005 (5)	−0.0010 (5)	0.0020 (5)
C12	0.0431 (7)	0.0437 (7)	0.0454 (7)	−0.0106 (6)	−0.0011 (6)	0.0032 (6)
C13	0.0550 (9)	0.0468 (8)	0.0499 (8)	−0.0182 (7)	−0.0099 (6)	0.0010 (7)
C14	0.0599 (9)	0.0446 (7)	0.0392 (7)	−0.0069 (7)	−0.0064 (6)	−0.0033 (6)
C15	0.0414 (7)	0.0355 (6)	0.0384 (7)	0.0047 (5)	−0.0030 (5)	0.0016 (5)
C16	0.0445 (7)	0.0547 (8)	0.0362 (7)	0.0053 (6)	0.0017 (5)	−0.0019 (6)
C17	0.0380 (7)	0.0662 (9)	0.0452 (8)	0.0001 (7)	0.0079 (6)	0.0039 (7)
C18	0.0328 (7)	0.0502 (8)	0.0504 (8)	−0.0046 (6)	0.0027 (6)	−0.0005 (6)

### Geometric parameters (Å, º)

**Table:**

O1—C3	1.3587 (14)	C9—C10	1.4230 (17)
O1—H1O	0.8200	C7—H7	0.9300
N1—C9	1.3865 (16)	C8—H8A	0.9600
N1—C1	1.4529 (17)	C8—H8B	0.9600
N1—H1N	0.870 (15)	C8—H8C	0.9600
C1—N2	1.4745 (16)	C10—C15	1.4161 (17)
C1—C2	1.5074 (17)	C10—C11	1.4168 (16)
C1—H1	0.9800	C11—C12	1.3744 (18)
O2—C4	1.3677 (15)	C12—C13	1.4017 (19)
O2—C8	1.4088 (17)	C12—H12	0.9300
N2—C11	1.4124 (16)	C13—C14	1.355 (2)
N2—H2N	0.864 (15)	C13—H13	0.9300
C2—C3	1.3922 (17)	C14—C15	1.4160 (19)
C2—C7	1.3930 (17)	C14—H14	0.9300
C3—C4	1.3942 (17)	C15—C16	1.4074 (18)
C4—C5	1.3826 (18)	C16—C17	1.359 (2)
C5—C6	1.386 (2)	C16—H16	0.9300
C5—H5	0.9300	C17—C18	1.397 (2)
C6—C7	1.370 (2)	C17—H17	0.9300
C6—H6	0.9300	C18—H18	0.9300
C9—C18	1.3710 (17)		
			
O1···O2	2.5772 (14)	C5···H8A^i^	2.94
O1···N2	2.6668 (14)	C5···H8B	2.72
C12···O1^i^	3.1736 (17)	C5···H8C	2.80
C17···O1^ii^	3.3145 (17)	C8···H5	2.55
C11···O1^i^	3.3650 (15)	H13···C9^i^	2.93
N2···O1^i^	2.9867 (14)	H13···C10^i^	2.97
H2N···O1^i^	2.196 (15)	C10···H1	2.95
H18···O1^ii^	2.88	C12···H1O^i^	2.88
H12···O1^i^	2.66	H1···H7	2.39
H17···O1^ii^	2.63	H1N···H18	2.44
O2···H6^iii^	2.87	H1O···H2N	2.31
N1···H1O	2.86	H17···H1O^ii^	2.57
H12···N1^i^	2.86	H18···H1O^ii^	2.47
N2···H1O	1.96	H2N···H12	2.43
C18···C12^ii^	3.567 (2)	H5···H8B	2.28
C1···H1O	2.46	H5···H8C	2.40
C4···H8A^i^	2.92	H14···H16	2.53
			
C3—O1—H1O	109.5	C2—C7—H7	119.5
C9—N1—C1	116.86 (10)	O2—C8—H8A	109.5
C9—N1—H1N	115.4 (10)	O2—C8—H8B	109.5
C1—N1—H1N	113.3 (10)	H8A—C8—H8B	109.5
N1—C1—N2	106.56 (10)	O2—C8—H8C	109.5
N1—C1—C2	109.17 (10)	H8A—C8—H8C	109.5
N2—C1—C2	111.38 (10)	H8B—C8—H8C	109.5
N1—C1—H1	109.9	C15—C10—C11	119.89 (11)
N2—C1—H1	109.9	C15—C10—C9	119.70 (11)
C2—C1—H1	109.9	C11—C10—C9	120.31 (11)
C4—O2—C8	117.50 (11)	C12—C11—N2	121.79 (11)
C11—N2—C1	115.25 (10)	C12—C11—C10	119.96 (11)
C11—N2—H2N	111.1 (10)	N2—C11—C10	118.20 (10)
C1—N2—H2N	110.1 (10)	C11—C12—C13	119.68 (12)
C3—C2—C7	118.64 (12)	C11—C12—H12	120.2
C3—C2—C1	121.17 (11)	C13—C12—H12	120.2
C7—C2—C1	120.00 (11)	C14—C13—C12	121.57 (13)
O1—C3—C2	122.54 (11)	C14—C13—H13	119.2
O1—C3—C4	116.99 (11)	C12—C13—H13	119.2
C2—C3—C4	120.47 (11)	C13—C14—C15	120.54 (12)
O2—C4—C5	125.78 (12)	C13—C14—H14	119.7
O2—C4—C3	114.47 (11)	C15—C14—H14	119.7
C5—C4—C3	119.75 (12)	C16—C15—C14	123.25 (12)
C4—C5—C6	119.84 (12)	C16—C15—C10	118.50 (12)
C4—C5—H5	120.1	C14—C15—C10	118.24 (12)
C6—C5—H5	120.1	C17—C16—C15	120.63 (13)
C7—C6—C5	120.39 (12)	C17—C16—H16	119.7
C7—C6—H6	119.8	C15—C16—H16	119.7
C5—C6—H6	119.8	C16—C17—C18	121.31 (13)
C18—C9—N1	123.65 (12)	C16—C17—H17	119.3
C18—C9—C10	119.61 (12)	C18—C17—H17	119.3
N1—C9—C10	116.62 (11)	C9—C18—C17	120.20 (13)
C6—C7—C2	120.90 (13)	C9—C18—H18	119.9
C6—C7—H7	119.5	C17—C18—H18	119.9
			
C9—N1—C1—N2	56.76 (13)	C18—C9—C10—C15	−0.81 (17)
C9—N1—C1—C2	177.15 (10)	N1—C9—C10—C15	−176.97 (10)
N1—C1—N2—C11	−52.57 (13)	C18—C9—C10—C11	175.67 (11)
C2—C1—N2—C11	−171.54 (10)	N1—C9—C10—C11	−0.50 (16)
N1—C1—C2—C3	−78.14 (14)	C1—N2—C11—C12	−157.13 (12)
N2—C1—C2—C3	39.26 (16)	C1—N2—C11—C10	25.13 (15)
N1—C1—C2—C7	96.72 (13)	C15—C10—C11—C12	1.88 (17)
N2—C1—C2—C7	−145.88 (12)	C9—C10—C11—C12	−174.59 (11)
C7—C2—C3—O1	−179.32 (11)	C15—C10—C11—N2	179.66 (10)
C1—C2—C3—O1	−4.39 (18)	C9—C10—C11—N2	3.19 (17)
C7—C2—C3—C4	0.17 (18)	N2—C11—C12—C13	179.45 (12)
C1—C2—C3—C4	175.10 (11)	C10—C11—C12—C13	−2.86 (19)
C8—O2—C4—C5	−2.9 (2)	C11—C12—C13—C14	0.8 (2)
C8—O2—C4—C3	176.72 (12)	C12—C13—C14—C15	2.2 (2)
O1—C3—C4—O2	−0.28 (16)	C13—C14—C15—C16	175.57 (13)
C2—C3—C4—O2	−179.80 (11)	C13—C14—C15—C10	−3.1 (2)
O1—C3—C4—C5	179.39 (12)	C11—C10—C15—C16	−177.68 (11)
C2—C3—C4—C5	−0.13 (18)	C9—C10—C15—C16	−1.19 (17)
O2—C4—C5—C6	179.60 (12)	C11—C10—C15—C14	1.09 (17)
C3—C4—C5—C6	0.0 (2)	C9—C10—C15—C14	177.58 (11)
C4—C5—C6—C7	0.2 (2)	C14—C15—C16—C17	−176.13 (13)
C1—N1—C9—C18	152.51 (12)	C10—C15—C16—C17	2.57 (19)
C1—N1—C9—C10	−31.50 (15)	C15—C16—C17—C18	−2.0 (2)
C5—C6—C7—C2	−0.1 (2)	N1—C9—C18—C17	177.36 (12)
C3—C2—C7—C6	−0.1 (2)	C10—C9—C18—C17	1.5 (2)
C1—C2—C7—C6	−175.04 (13)	C16—C17—C18—C9	−0.1 (2)

Symmetry codes: (i) −*x*+3/2, *y*+1/2, *z*; (ii) *x*−1/2, *y*, −*z*+3/2; (iii) *x*+1/2, −*y*+1/2, −*z*+1.

### Hydrogen-bond geometry (Å, º)

*Cg*1 and *Cg*4 are the centroids of rings *A* (C2–C7) and *D* (C9/C10/C15–C18), respectively.

**Table:**

*D*—H···*A*	*D*—H	H···*A*	*D*···*A*	*D*—H···*A*
O1—H1*O*···N2	0.82	1.96	2.6667 (14)	144
N2—H2*N*···O1^i^	0.864 (15)	2.196 (15)	2.9870 (14)	152.2 (13)
C8—H8*A*···*Cg*1^iv^	0.96	2.82	3.6580 (17)	146
C13—H13···*Cg*4^i^	0.93	2.87	3.7336 (16)	155
C16—H16···*Cg*1^v^	0.93	2.87	3.4880 (15)	125

Symmetry codes: (i) −*x*+3/2, *y*+1/2, *z*; (iv) *x*, −*y*−1/2, *z*−1/2; (v) *x*+1/2, −*y*−1/2, −*z*.
